# Functional Aerogels Composed of Regenerated Cellulose and Tungsten Oxide for UV Detection and Seawater Desalination

**DOI:** 10.3390/gels9010010

**Published:** 2022-12-25

**Authors:** Yanjin Tang, Yuhan Lai, Ruiqin Gao, Yuxuan Chen, Kexin Xiong, Juan Ye, Qi Zheng, Zhenxing Fang, Guangsheng Pang, Hoo-Jeong Lee

**Affiliations:** 1 College of Science and Technology, Ningbo University, 521 Wenwei Road, Ningbo 315300, China; 2 Department of Smart Fab. Technology, Sungkyunkwan University, Suwon 16419, Republic of Korea; 3 School of Biological and Chemical Engineering, NingboTech University, No. 1 South Qianhu Road, Ningbo 315100, China; 4 State Key Laboratory of Inorganic Synthesis and Preparative Chemistry College of Chemistry, Jilin University, Changchun 130012, China

**Keywords:** functional aerogels, regenerated cellulose, color center, solar absorbent, desalination

## Abstract

Functional aerogels composed of regenerated cellulose and tungsten oxide were fabricated by implanting tungsten-oxide nanodots into regenerated cellulose fiber. This superfast photochromic property benefitted from the small size and even distribution of tungsten oxide, which was caused by the confinement effect of the regenerated cellulose fiber. The composite was characterized using XRD and TEM to illustrate the successful loading of tungsten oxide. The composite turned from pale white to bright blue under ambient solar irradiation in five seconds. The evidence of solar absorption and electron paramagnetic resonance (EPR) demonstrated the fast photochromic nature of the composite and its mechanism. Furthermore, carbon fiber filled with preferential growth tungsten-oxide nanorods was obtained by annealing the photochromic composite in a N_2_ atmosphere. This annealed product exhibited good absorption across the whole solar spectrum and revealed an excellent photothermal conversion performance. The water evaporation rate reached 1.75 kg m^−2^ h^−1^ under one sun illumination, which is 4.4 times higher than that of pure water. The photothermal conversion efficiency was 85%, which shows its potential application prospects in seawater desalination.

## 1. Introduction

Developing advanced building materials with both excellent thermal insulating and tunable optical properties to replace common glass is highly desirable for improving humans’ quality of life and reducing global energy consumption. Nanocellulose is a major composite of plants, whose resource is abundant on Earth and has been used in heat insulation by reflecting solar light [1]. Furthermore, nanocellulose is a promising dispersion material because of its outstanding advantages, such as being environmentally friendly, having a good film-forming property, and being a good dispersant for transition-metal oxide. Tungsten oxide has been widely used in the electrochromic and photochromic fields due to its unique crystal structure and electronic energy band structure [2,3]. The colored tungsten oxide can adsorb part of the visible light and near-infrared light, which has a potential application for cooling down the inside temperature, photothermal therapy, and catalysis [4,5,6,7]. The chromic nature of tungsten oxide is caused by the formation of a color center (reduced tungsten state W^5+^), occurring in both the electrochromic and photochromic processes [8,9,10]. The electrochromic process has two parts, namely coloring and bleaching, which represent the macroscopic appearance of the cation intercalation and deintercalation processes [11]. The reduced tungsten state can also be formed by the self-trapping of the photon caused by exposure to UV light, i.e., the photochromic effect [12,13]. The reduced tungsten state can dramatically increase the light absorption of the visible range and even the near-infrared range, which has been applied in photothermal conversion and NIR shielding [14,15,16,17]. However, the slight color change, slow response, and poor irreversibility of WO_3_ hinder its practical application. In general, photochromic efficiency has largely been limited by the separation and recombination of photon-induced electrons and holes. Thus, decreasing the particle size seems to be an effective way to reduce the migration path of the photon, which leads to an increase in photochromic efficiency. However, nanoparticles, even QDs, tend to aggregate due to the large surface energy, which hampers photochromic efficiency. Appling sub-nanoporous silica as a template has been reported to fabricate tungsten-oxide quantum dots (QDs) [18]. The spatial confinement effect of this porous silica prevents the WO_3_ QDs from aggregating into large particles. Suzuko et al. increased the photochromic efficiency of tungsten oxide by using glyceric acid as a dispersant to accelerate electron diffusion. However, another disadvantage (suppressed bleaching process) emerged due to the low oxygen diffusion [9]. Lacking effective contact with oxygen prolonged the bleaching time of the colored tungsten oxide. In this research, nanocellulose was chosen as the dispersant because of its abundant OH and COOH functional groups, which have also been widely used as an anti-quenching agent by preventing the fluorescent nanoparticles from assembling [19]. The excellent dispersion of tungsten oxides on the cellulose surface provides a fast solar response and bleaching ability without solar irradiation. Furthermore, the obtained cellulose filled with tungsten-oxide quantum dots changes into a carbon fiber filled with preferential growth tungsten-oxide nanorods, which exhibits good solar absorption and excellent photothermal conversion performance. The annealed composites can be applied in many areas, such as photothermal evaporation membranes, NIR shielding, photothermal therapy, etc. [20,21]. This excellent photothermal conversion performance also benefits from the reduced tungsten oxide (W^5+^) and the size, morphology, and orientation of the tungsten-oxide nanorods. Guo reported that nanorods may exhibit transverse and longitudinal surface plasmon resonances, which correspond to the electron oscillations perpendicular and parallel to the rod length direction, respectively.

## 2. Results and Discussion

**Figure 1:**
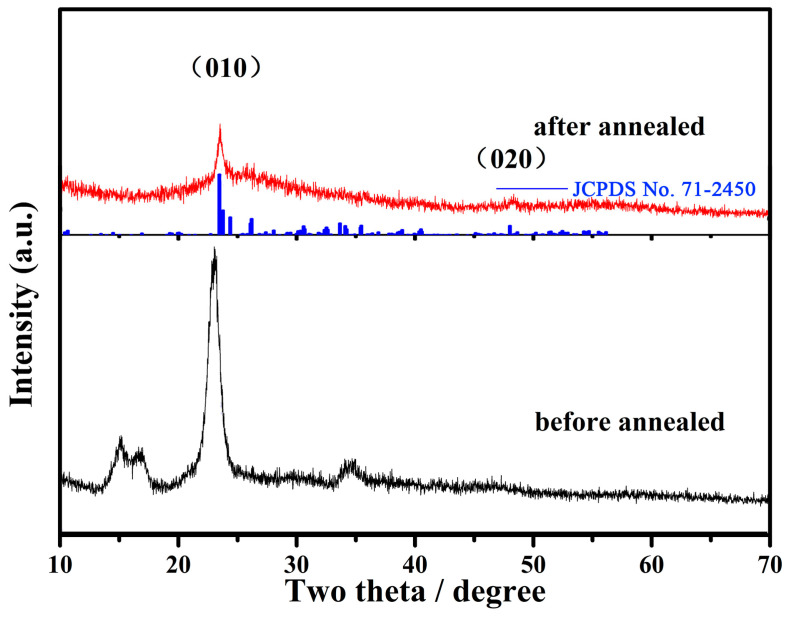
XRD patterns of the composite before and after heat treatment.

**Figure 2:**
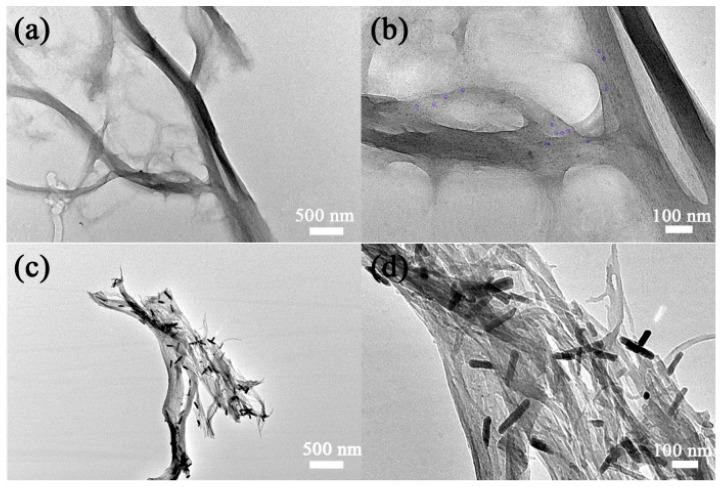
(**a**,**b**) TEM images of the composite before heat treatment. (**c**,**d**) TEM images of the composite after being annealed at 500 °C under N_2_.

Figure 1 shows the XRD patterns of the WO_3-x_@-regenerated cellulose fiber composites. As known from the Debye–Scherrer equation, the broad diffraction peaks in the black line revealed the small nanoparticle size of the tungsten oxides. This was caused by the confinement of the regenerated cellulose fiber during the tungsten-oxide crystal growth process. As we can see in Figure 2, the size of the regenerated cellulose was hundreds of nanometers in diameter, which was composed of several nanocellulose fibers. When the alcoholysis intermediate product penetrated into the regenerated cellulose fiber, it was confined in the gaps between the cellulose fibers. Therefore, this confinement effect benefited from the gaps generated during the formation of the nanocellulose fiber in the regenerated cellulose fiber. This confinement guaranteed the small size of the tungsten-oxide crystal in the hydrolysis reaction of the alcoholysis intermediate product. This result is also evidenced by the partially enlarged TEM image shown in Figure 2b. As for the diffraction results of the annealed sample (the red line), there were only two obvious diffraction peaks, indexed to the (010) and (020) crystal face, which were well matched with JCPDS No. 71-2450. The disappearance of other diffraction peaks was caused by the oriented growth along with the <010> direction when it was annealed in an inert atmosphere. This was also seen in other reported works [22,23]. Tungsten oxide is a well-known semiconductor because of its varied crystal structure and affordability of oxygen deficiency. The WO_6_ octahedron as the unit of the tungsten-oxide crystal structure presented a different arrangement under different temperatures. The crystal structure of tungsten oxide has a temperature-related nature. Furthermore, when being annealed under an inert atmosphere, oxygen deficiencies can be formed at the surface of the crystal structure, which has a large impact on the crystal structure. Thus, tungsten oxide always reveals preferential growth under an inert atmosphere at high temperatures. Therefore, the appearance of two diffraction peaks of parallel crystal faces showed that the tungsten oxides were loaded into the regenerated cellulose fiber. This is also evidenced by the enlarged TEM image shown in Figure 2d.

The morphology of the composite is shown in Figure 2. The morphology of the regenerated cellulose fiber with several um in length and a radius less than 100 nm can be seen in Figure 2a. This benefitted from the freezing and melting cycle process, which introduced ice crystals into the cellulose fibers to widen the distance between the fibers. This is a common way to decrease the hydrogen bonds between the cellulose fibers to induce a stable process of cellulose dispersion. As shown in the partially enlarged TEM image in Figure 2b, a large number of nanodots were anchored in the cellulose fiber. It is hard to see any small particles except in the gaps between the cellulose fibers. This result illustrates that the regenerated cellulose fiber acted as not only a growth substrate but also a confinement cage. These results were also consistent with the broad diffraction peaks of XRD diffraction shown with the black line. As shown in Figure 2c,d, these tungsten-oxide nanodots were transformed into nanorods after being annealed in a N_2_ atmosphere. This correlated with the XRD result of the red line diffraction pattern. Tungsten oxide prioritized growth along the <010> direction during the heat treatment under an inert atmosphere. Furthermore, the regenerated cellulose fiber was carbonized into carbon fiber during the annealing process. The carbonization process inevitably causes shrinkage, which can be seen by comparing Figure 2a,c and Figure 2b,d (taken at the same magnification). The tungsten-oxide nanodots confined in the regenerated cellulose fiber were closer and contacted each other during the cellulose shrinkage, which enabled the nanodots’ migration and the crystal’s preferential growth. The orientational growth of the tungsten-oxide nanorods was also anchored in the carbon fibers. It is difficult to see any nanorods except in the gaps between the carbon fibers (Figure 2c,d). This is very important for its practical application, which effectively prevents the loss of active material.

**Figure 3:**
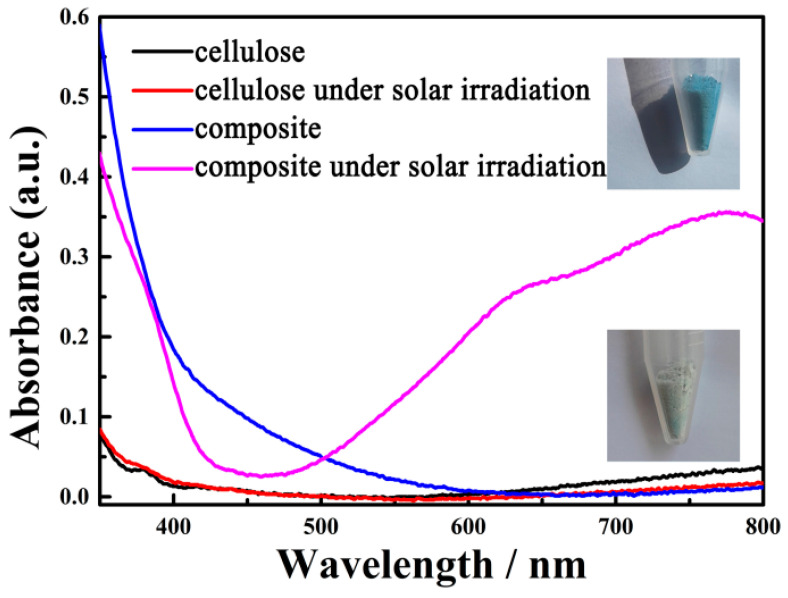
UV–Vis absorption spectra of the photochromic WO_3-x_@-regenerated cellulose fiber composite.

**Figure 4:**
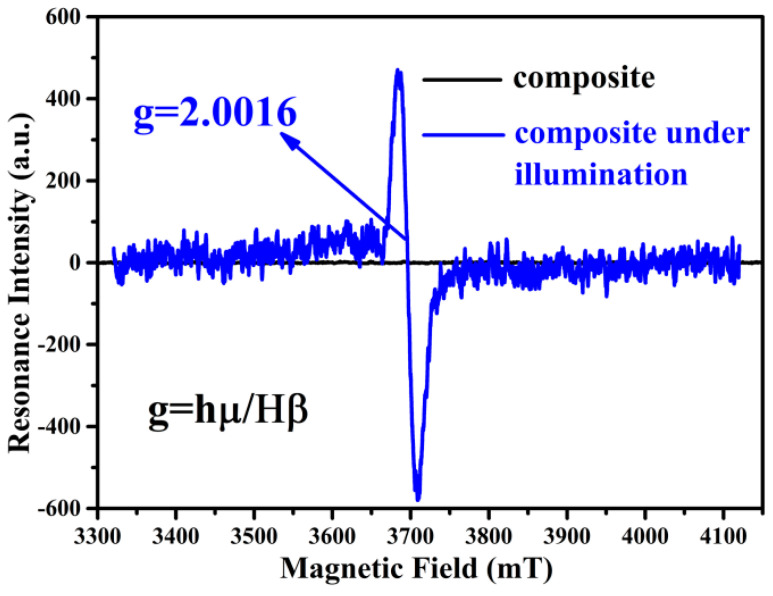
EPR spectra of the WO_2.72_@-regenerated cellulose composite under natural conditions (black) and illuminated by AM 1.5 (blue).

As discussed above, the tungsten-oxide nanodots were exactly located in the regenerated cellulose fiber; thus, the composite should have a good photochromic performance. As shown in Figure 3, the black and red lines at the bottom are the absorption spectra of the regenerated cellulose fiber before and after solar irradiation, respectively. It should be noted that this solar absorption spectrum is collected on solid films, not solutions, because of its low dispersion in water. The absorption of cellulose had almost no change at all after solar irradiation throughout the UV–Vis range. However, the composite of the WO_3-x_@-regenerated cellulose fiber exhibited a fast photochromic property. As the inset pictures reveal, the appearance of the composite turned from pale white to bright blue under ambient solar irradiation; the absorption intensity of the composite increased up to 0.3 from 0.01 during the long wavelength range from 600 to 800 nm. This fast photochromic performance can be attributed to the small particle size of tungsten oxide, just as Figure 2b shows. The appearance of the WO_3-x_@-regenerated cellulose fiber composite quickly turned blue from pale in less than 10 s under solar irradiation (the photochromic process can be seen in the Supplemental Material Video S1). This photochromic nature was caused by a photon-induced electron self-trapping process, which introduced the formation of the color center W^5+^. Furthermore, the formation of the color center was verified with the EPR experiment. The single electron in the 4f orbital of W^5+^ was detected by the fluctuating magnetic field. The signal peak in Figure 4 was exactly caused by the single-electron spinning resonance, which was evidenced by the value of the g factor (the same as that of the free radical). This result revealed that the composite could generate a large amount of W^5+^ under illumination by AM 1.5. This fast photochromic property came from the evenly distributed tungsten-oxide nanodots, which dramatically decreased the carrier migration distance so that the process of the carriers’ recombination was depressed. Due to the surrounding regenerated cellulose fiber, the surface area of the tungsten-oxide dots exposed to contact with oxygen in the air was decreased. This is critical to the photochromic nature of this composite. However, the WO_3-x_@-regenerated cellulose fiber composite turned back to pale white when in contact with the air for a period of time without solar irradiation, which demonstrated its good circularity. Thus, this product might have some potential applications in window films.

**Figure 5:**
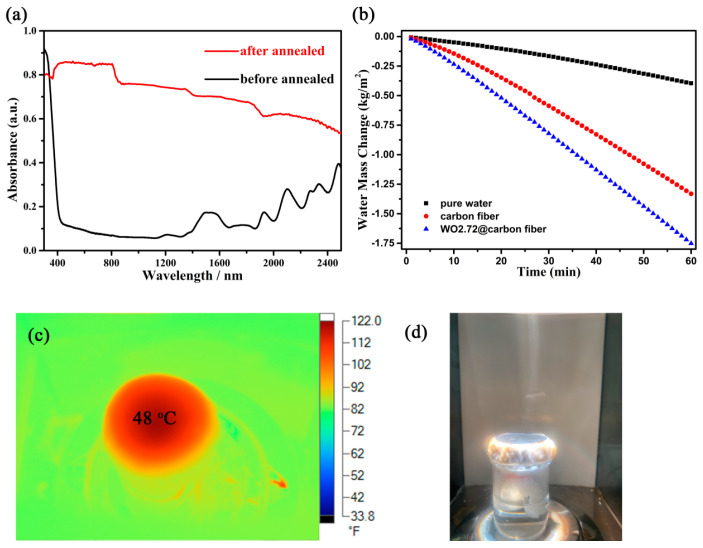
(**a**) Solar absorption spectrum of the cellulose fiber and the WO_2.72_@carbonized cellulose fiber. (**b**) Water evaporation test of the WO_2.72_@carbon fiber. (**c**) The photothermal image of the WO_2.72_@carbon fiber under one sun irradiation. (**d**) Digital image of the evaporation for the WO_2.72_@carbon fiber.

As Figure 2c,d shows, the orientational growth of the nanorods was anchored in the carbonized fiber. The nanorod morphology depressed the light scattering to a large extent. The high concentration of free carriers in the semiconductor coupled with the carbonized cellulose fiber had excellent solar absorption, as shown in Figure 5a. As is known, the amorphous carbon material only absorbs visible light, not near-infrared light. Only some graphene-based carbon materials have a small near-infrared absorption capability due to their single electron on each carbon atom. Apparently, the carbon fiber obtained in this work could not be graphitized at the low carbonization temperature without catalysts. Therefore, the good absorption between 800 and 2500 nm of the annealed product (red line) mostly came from the oxygen deficiencies in WO_2.72_. A new orbital energy level was introduced into the valance and conductive band when oxygen vacancies formed in the semiconductor. The collective oscillations of the surface free carriers (electron for n-type semiconductor WO_2.72_) induced the surface plasmon resonance, which indicated a good photothermal conversion performance. As for the cellulose before being annealed, the weak absorption in the range from 1600 to 2500 nm was due to its surface functional groups such as COOH and OH, which is a common occurrence in biomass. Therefore, a water evaporation test was conducted under one sun illumination to evaluate the photothermal conversion performance. The results are shown in Figure 5b; the water evaporation rate of the WO_2.72_@carbon fiber reached 1.75 kg m^−2^ h^−1^, which was faster than that of carbonized fiber and 4.4 times higher than that of pure water (0.39 kg m^−2^ h^−1^). The water evaporation efficiency of the carbonized cellulose fiber was 1.33 kg m^−2^ h^−1^, which was lower than that of the graphene-based carbon materials [24]. This was determined by its electronic structure. The free-carrier concentration of the carbon fiber was very low, which could not effectively absorb the low-frequency photons to generate heat. According to the calculation equation of light to heat for water evaporation efficiency [25], the conversion efficiency of the WO_2.72_@carbon fiber was 85%. This evaporation efficiency was higher than that of the reported 2D evaporators [26,27,28]. It should be mentioned that 2D evaporators aim to evaluate the photothermal conversion performance, and 3D evaporators aim to increase the water evaporation rate. Therefore, the water evaporation rate was limited by the finite evaporation surface. Here, water evaporation consumed a large amount of the heat generated by the photothermal conversion membrane to realize the phase transition; even with this, the surface temperature still reached 48 °C. Therefore, the composite of the WO_2.72_@carbon fiber revealed an excellent photothermal conversion performance. As shown in Figure 5d, above the evaporation surface of the WO_2.72_@carbon fiber membrane, the steam generation under one sun irradiation (simulated solar light irradiation, whose power was 100 mW/cm^2^) was clear. The dynamic evaporation process recorded by a cell phone can be seen in Supplemental Material Video S2. Therefore, the photothermal evaporation membrane fabricated with the assistance of vacuum filtration might have a promising application in the solar desalination field.

## 3. Conclusions

A superfast photochromic material was obtained by implanting tungsten-oxide nanodots into regenerated cellulose fiber. This superfast photochromic property benefitted from the small size and even distribution of the tungsten oxide, which was caused by the confinement effect of the regenerated cellulose fiber. This reversible photochromic nature shows promising applications for UV detectors, smart windows, NIR shielding, etc. The WO_2.72_@carbon fiber composite had a broad and strong absorption across the whole solar spectrum. The anchoring effect of the substrate on the photothermal conversion material guarantees its long-term use. Its excellent photothermal conversion performance also makes it a promising application for solar desalination.

## 4. Materials and Methods

The materials described in this report were all purchased from Aladdin. There was no need to purify before use. All the materials were Analytical Reagents. The purity of NaOH was higher than 96%. The purity of urea and WCl_6_ was 99%, and the purity of n-butanol and DMF was 99.5%.

### 4.1. Synthesis of the Regenerated Cellulose Fiber

The preparation solvent was as follows: First, an amount of 10 g NaOH was dissolved in 90 mL of deionized water to obtain a 10 wt% NaOH solution. Then, 0.9 g urea was added to decrease the polarity of the solution (prevent cellulose excessive dissociation). Next, 1 g of filter paper scraps was immersed into the mixture of NaOH and urea solution, followed by three repeated programs of freezing at −18 °C and melting at room temperature. The freezing process lasted at least 4 h to ensure the mixture was completely frozen. The regenerated cellulose fiber was obtained by centrifuging the mixture, followed by a vacuum-assisted drying process.

### 4.2. Synthesis of the WO_3-x_@-Regenerated Cellulose Fiber

First, 100 mg WCl_6_ and 1 g of the regenerated cellulose fiber were dispersed in 60 mL of n-butanol and DMF via sonication. This light-yellow dispersion was transferred into a 100 mL stainless autoclave and kept at 200 °C for 24 h. After cooling down to room temperature, the pale-white precipitate was centrifuged and washed with deionized water and ethanol three times. The WO_3-x_@-regenerated cellulose fiber composite was dried in a vacuum oven at 60 °C overnight.

### 4.3. Synthesis of the WO_2.72_@Carbon Fiber

The obtained WO_3-x_@-regenerated cellulose fiber was annealed in a high-purity N_2_ environment at 500 °C for 2 h with a 10 °C /min ramping rate. After cooling down to room temperature, dark black powders were obtained. The WO_2.72_@carbon fiber was dispersed in DMF via sonication to induce stable dispersion. The photothermal evaporation membrane was fabricated with the assistance of vacuum filtration.

### 4.4. Characterization

The XRD patterns were recorded using PANalytical B.V. Empyrean X-ray powder diffraction with Cu Kα radiation over a range of 10–70° (2θ) with 0.02° per step. The transmission electron microscope (TEM) images were obtained with a Tecnai G2 FEI electron microscope. UV–vis adsorption spectra were recorded using a Lambda 950 spectroscope. Electron paramagnetic resonance (EPR) spectra were obtained with a JES-FA 200 EPR spectrometer. The EPR test of the colored composite was conducted under simulated light irradiation. The solar absorption spectrum was recorded with a Shimadzu UV-2450 spectroscope across the whole solar spectrum (200–2500 nm). The photothermal image was filmed using Fluke Ti 32s. The water-mass change was recorded with an electrical balance (FA 2004) under one sun irradiation (AM 1.5).

## Supplementary Materials

The following supporting information can be downloaded at: https://www.mdpi.com/article/10.3390/gels9010010/s1, Video S1: photochromic; Video S2: evaporation.
